# Disease as an Accident

**Published:** 1914-03-14

**Authors:** 


### LEGAL NOTES FOR INSTITUTIONAL WORKERS.

### Disease as an Accident.

Of all the varied questions which the Workmen's Compensation Act has raised, the finest and most difficult of solution has certainly been the question whether or not, and when, the contraction of a disease constitutes an accident. Having recently had to consider the matter, we think a statement of what guiding principles we may have found, with illustrations, will be of interest.

Generally speaking, in order to constitute an accident the disease must be one the contraction of which can be definitely fixed in point of time as an event. For instance, a workman was employed to open and sort bales of Persian wool. While so engaged his eye became infected with anthrax, which necessitated an operation, from which he died. The disease was caused by a bacillus alighting on his eye. In this instance it could be told definitely the day on which the injury occurred and with considerable certainty the manner in which it occurred, and it was, therefore, held to be due to an accident. And similarly in another anthrax case, in which the disease was contracted through a pimple on the workman's neck, it was said by the Master of the Rolls (Collins): "If a workman dies of or is injured by a disease which he himself has brought with him into his work, how could he be said to die from an 'accident'? But that is very different from a case where a workman accidentally catches an infection in the course of his employment. The disease here was caused by the attack or incursion of some bacillus or germ. Though the attack was infinitesimal in force and invisible to the naked eye, yet it was physically a blow, the incursion of a physical force which seems to come well within the words used by Lord Macnaghten in describing the sense in which he thinks the word 'accident' is used in the statute."

The contraction of a cold or chill may amount to an accident, depending upon the circumstances of the instant case. While employed in clearing a mill-race a workman caught a sudden chill, caused by immersion in the water. Inflammation of the kidneys supervened, and he died several days later. The evidence showed that the attack could only have been brought on by exposure to cold water. It was held that the death was due to an accident.

Sunstroke or heatstroke may constitute an accident within the meaning of the Workmen's Compensation law. A workman of poor physique was employed in the stokehold of a vessel. The conditions there were normal, but, as usual, the place was very warm. The man suffered a heatstroke which resulted in his death. It was held that his death was due to an accident. It should be mentioned, however, that in an earlier English case arising under an insurance policy it was held that a sunstroke received by the master of a vessel then sailing in the tropics was not an accident; and the American Courts have held that sunstroke contracted by a supervising architect in the course of his ordinary duties was not an injury by accidental means, that being a result flowing ordinarily and naturally from the conduct of the person, although he may not have foreseen the consequences.

It has not been definitely decided under any Workmen's Compensation Act whether frost-bite is injury by accident. In Warner *v.* Couchman (1911, 1 K.B., 351) it was admitted for the sake of argument that it did amount to an accident in that case, but Cozens Hardy, M.R., said that he felt considerable doubt whether there was an accident.

Death from heart disease may or may not be from accident. The question depends for its solution upon the circumstances of the given case. Death brought on by the gradual progress of the disease is not due to an accident. But death or injury from heart disease caused by a sudden strain may well be considered so.

### A Question of Fact and Law.

A workman was descending the side of a ship by means of a rope ladder when the ladder twisted suddenly, and with a cry he fell into the water. Three minutes later he was picked up dead. The evidence showed that death was due to heart failure, and probably occurred before he reached the water. It was further shown that his heart was in a bad state, that descending the ladder would cause a strain, that the sudden twisting of the ladder would be likely to bring on heart failure, and that the mere exertion of walking uphill or coughing or sneezing might have been fatal to him. The County Court Judge found that death was due to an accident, and on appeal it was held that there was evidence to support the finding. On the other hand, where a man who has been suffering for several years from progressive heart disease became faint while hurrying to a railway station with a parcel for his employer, and died shortly afterwards, it was held that his death was due to disease, not accident.

Other illustrations might be given, but the above are typical. The Courts consider the matter of whether or not an accident took place as one of fact, but really and in practice it is one of mixed fact and law. When the facts are found it is then a question of law whether they constitute an accident.

